# Reduced cerebral cortex thickness is related to overexpression of exosomal miR-146a-5p in medication-free patients with major depressive disorder

**DOI:** 10.1017/S0033291722003567

**Published:** 2023-10

**Authors:** Yanjia Deng, Ping Gong, Shuguang Han, Jingyu Zhang, Shuai Zhang, Bin Zhang, Yong Lin, Kai Xu, Ge Wen, Kai Liu

**Affiliations:** 1 School of Medical Imaging, Xuzhou Medical University, Xuzhou, China; 2 Department of Radiology, Affiliated Hospital of Xuzhou Medical University, Xuzhou Medical University, Xuzhou, China; 3 Medical Imaging Department, Nanfang Hospital, Southern Medical University, Guangzhou, China; 4 Department of Psychiatry, Nanfang Hospital, Southern Medical University, Guangzhou, China; 5 The fifth affiliated hospital of Sun-Yat Sen University, Sun-Yat Sen University, Zhuhai, China; 6 The Affiliated Brain Hospital of Guangzhou Medical University (Guangzhou Huiai Hospital), Guangzhou, China

**Keywords:** Cortical thickness, magnetic resonance imaging, major depressive disorder, MicroRNA

## Abstract

**Background:**

Previous studies have confirmed that miR-146a-5p overexpression suppresses neurogenesis, thereby enhancing depression-like behaviors. However, it remains unclear how miR-146a-5p dysregulation produces *in vivo* brain structural abnormalities in patients with major depressive disorder (MDD).

**Methods:**

In this case–control study, we combined cortical morphology analysis of magnetic resonance imaging (MRI) and miR-146a-5p quantification to investigate the neuropathological effect of miR-146a-5p on cortical thickness in MDD patients. Serum-derived exosomes that were considered to readily cross the blood-brain barrier and contain miR-146a-5p were isolated for miRNA quantification. Moreover, follow-up MRI scans were performed in the MDD patients after 6 weeks of antidepressant treatment to further validate the clinical relevance of the relationship between miR-146a-5p and brain structural abnormalities.

**Results:**

In total, 113 medication-free MDD patients and 107 matched healthy controls were included. Vertex-vise general linear model revealed miR-146a-5p-dependent cortical thinning in MDD patients compared with healthy individuals, i.e., overexpression of miR-146a-5p was associated with reduced cortical thickness in the left orbitofrontal cortex (OFC), anterior cingulate cortex, bilateral lateral occipital cortices (LOCs), etc. Moreover, this relationship between baseline miR-146a-5p and cortical thinning was nonsignificant for all regions in the patients who had received antidepressant treatment, and higher baseline miR-146a-5p expression was found to be related to greater longitudinal cortical thickening in the left OFC and right LOC.

**Conclusions:**

The findings of this study reveal a relationship between miR-146a-5p overexpression and cortical atrophy and thus may help specify the *in vivo* mediating effect of miR-146a-5p dysregulation on brain structural abnormalities in patients with MDD.

## Introduction

Previous studies have demonstrated that microRNA-146a-5p (miR-146a-5p) suppresses neurogenesis and is strongly associated with depressive phenotypes in both animal models and patients with depression (Fan et al., 2022; Lopez et al., 2017a; Prada et al., 2018). MiR-146a-5p is a microglia-specific neuronal miRNA (Prada et al., 2018). Its overexpression is considered to affect excitatory synapses (McNeill & Van Vactor, 2012) and mediate neuroinflammatory responses (Prada et al., 2018) and thus may be involved in the pathogenesis and progression of major depressive disorder (MDD) (Wohleb, Franklin, Iwata, & Duman, 2016). For example, Prada et al., revealed that microglia transport miR-146a-5p to neurons via extracellular vesicles (EVs), thereby controlling the expression of presynaptic synaptotagmin1 and postsynaptic neuroligin1 (both of which play important roles in spine formation and synaptic stability) (Prada et al., 2018). Morphological analysis further revealed that prolonged exposure to EVs with miR-146a-5p leads to dendritic spine loss and a decrease in the density and strength of excitatory synapses (Prada et al., 2018).

Regarding pathway-level findings specifically, it has been reported that miR-146a-5p can regulate the Krüppel-like factor 4 (KLF4)/cyclin-dependent kinase-like 5 (Cdkl5) pathway and further influence neurogenesis. In particular, overexpression of exosomal miR-146a-5p decreases KLF4 (Cui et al., 2017) expression in neural stem cells and thus regulates the differentiation and migration of neurons (Cui et al., 2017). In addition, miR-146a-5p can bind to KLF4 in order to inhibit the expression of Cdkl5, thereby inhibiting the formation of new granular neurons and ultimately reducing neurogenesis in depression (Fuchs et al., 2014). In Lopez's study based on small RNA sequencing, miR-146a-5p was found to be one of the three consistent markers of antidepressant response and regulators of the MAPK/Wnt systems across clinical trials, animal models, and postmortem studies (Lopez et al., 2017a). In fact, miR-146a-5p participates in the regulation of 430 genes involved in the MAPK/Wnt signaling pathway, and most of these genes have been previously reported to be associated with MDD or antidepressant activity (Lopez et al., 2017a). In particular, under regulatory control by miRNA-146a-5p, tropomyosin receptor kinase B (NTRK2), as the preferred receptor of brain-derived neurotrophic factor (BDNF), plays a key role in the neurotrophic hypothesis of depression (Chao, 2003; Lopez et al., 2013). Taken together, these findings confirm the role of miR-146a-5p dysregulation in the neuropathology of MDD and suggest a relationship between miR-146a-5p and neuroanatomical abnormalities in patients (e.g. gray matter atrophy related to inhibited neurogenesis). Unfortunately, only a few studies to date have investigated the *in vivo* effect of epigenetic regulators in the brains of patients with MDD (He et al., 2021; Lopez et al., 2017b). The specific *in vivo* mechanism by which dysregulated miR-146a-5p mediates structural abnormalities in the depressed brain and ultimately contributes to depressive behavior remains to be elucidated.

Peripheral blood is one of the most accessible, noninvasive sources of genetic information in the human body, but it may not be representative of the microenvironment of the central nervous system (CNS) because they are separated by the blood-brain barrier (BBB). Exosomes are thought to readily cross the BBB, making brain-derived exosomes easily accessible via peripheral blood sampling. Neuronal miRNA expression is a highly cell-type-specific process (Jørgensen et al., 2009). More importantly, a previous study demonstrated that the miR-146a-5p derived from serum exosomes in a depressed rat model was mainly of neural origin (Fan et al., 2022). In particular, miR-146a-5p-containing exosomes derived from serum were mainly secreted by microglia based on the common expression of microglia-specific markers (CD13, CD14, MCT-1, etc.) (Potolicchio et al., 2005). Therefore, peripheral blood exosomes carrying CNS-relevant genetic information of miR-146a-5p from a functioning brain yield an ideal candidate for *in vivo* imaging-genetic investigation.

In this study, we combined magnetic resonance imaging (MRI)-based analysis of cortical morphology with assessment of serum exosomal miR-146a-5p to investigate the neuropathological effect of miR-146a-5p on cortical gray matter in MDD patients. We hypothesized that miR-146a-5p dysregulation could mediate differences in cortical characteristics (e.g. cortical atrophy in specific regions) in depressed patients compared with healthy controls (HCs) and could ultimately be related to the pathogenesis of depression. Moreover, after 6 weeks of antidepressant treatment, follow-up data were collected to identify whether the underlying pathological relationship between miR-146a-5p and brain structural abnormalities in MDD patients could be changed (e.g. prevented or even reversed) via treatment, with the goal of further validating the pathological and clinical relevance of the results. We hope the findings will help to shed light on the interplay among microRNAs, the brain, and behavior.

## Materials and methods

### Participants

This study is registered in the Chinese Clinical Trial Registry (http://www.chictr.org.cn, No. ChiCTR1800017780). Before the study, each participant was informed of all procedures for the experiment and signed a written informed consent form. Ethical approval was obtained from all the institutions involved in data collection. Data collection in this study followed a case–control design. Patients diagnosed with MDD (the MDD group) and HCs (the HC group) were recruited from the outpatient department and through advertisements in Guangzhou, China, from April 2018. Healthy individuals were included as controls after they were frequency-matched to MDD patients at a ratio of 1:1 according to age, sex, and education.

The inclusion criteria were as follows (criteria 1 and 2 apply only to the MDD group, criterion 3 applies only to the HC group only, and all other criteria apply to both groups): (1) first-episode, drug-naïve MDD or recurrent MDD that met the diagnostic criteria for MDD according to the Structured Clinical Interview for the Diagnostic and Statistical Manual of Mental Disorders (DSM)-IV; (2) a 24-item Hamilton Rating Scale for Depression (HAMD) score ⩾ 17; (3) the absence of any psychiatric disorders according to the DSM-IV; (4) age between 18 and 65 years; (5) right-handedness; (6) Han-Chinese ancestry; and (7) agreement to participate after being informed about the study.

The exclusion criteria were as follows (criterion 1 applies only to the MDD group, and all other criteria apply to both groups): (1) history of any other psychiatric disorder (e.g. schizophrenia); (2) secondary depression caused by drugs or organic diseases; (3) use of psychotropic medications (including antidepressants, mood stabilizers, antipsychotics, stimulants, sedative hypnotics, and benzodiazepines) in the 8 weeks before MR scanning and blood sampling; (4) electroconvulsive therapy within the past 6 months; (5) any history of substance abuse or dependence (excluding nicotine); (6) history of severe suicidal behavior; (7) any contraindication to MR scanning; (8) any organic lesion in the brain; (9) history of head trauma with loss of consciousness > 10 min; and (10) documented intellectual impairment. To reduce disturbances in peripheral blood caused by known clinical complications (e.g. inflammation, endocrine dysfunction, use of medication), we additionally excluded participants with (11) long-term use of medications known to affect the immune system (e.g. nonsteroidal or steroidal anti-inflammatory drugs, statins/angiotensin 2 receptor inhibitors), (12) hepatorenal/cardiopulmonary dysfunction or endocrine disease; (13) any evidence of active infection (e.g. fever, increased blood C-reactive protein or elevated white blood cell count) within 1 week before data collection; and (14) any history of chronic disease relating to a chronic inflammatory status (specifically, chronic hepatitis, nephritis, pulmonary diseases, or autoimmune disorders).

To identify the influence of antidepressant treatment on the possible relationship between miR-146a-5p and cortical thickness, we additionally performed a longitudinal observation with antidepressant treatment. After initial inclusion, a cohort of MDD patients voluntarily participated in the subsequent 6-week follow-up, during which they were naturalistically treated with selective serotonin reuptake inhibitors, including paroxetine, sertraline, and escitalopram. The dosage [ranging from 11.11 to 70.59 mg/day in fluoxetine equivalents (Hayasaka et al., 2015)] and type of medication were assigned to each patient according to the experience of the psychiatrist and the physical status of the patient. At the end of the 6-week treatment period, a second MR scan was performed.

### Isolation and verification of circulating exosomes

See online Supplementary Appendix 1 and Table S1 for details.

### RNA extraction and quantification

See online Supplementary Appendix 2 for details.

### MRI acquisition

To avoid treatment delay, MR scans for all patients were completed within 24 h after enrollment. To avoid bias during MR data collection, the MR scans for all the subjects were performed by the same technician with a single MR scanner (Achieva 3.0 T TX, Philips Medical Systems, Best, The Netherlands) and a quadrature head coil. Three-dimensional T1-weighted (3D-T1W) images were acquired using a turbo field-echo sequence with the following parameters: time of repetition (TR) = 8.2 ms, time of echo (TE) = 3.8 ms, flip angle = 7°, bandwidth = 191 Hz, field of view (FOV) = 256 × 256 mm^2^, voxel size = 1.0 × 1.0 × 1.0 mm^3^.

### MR image preprocessing

See online Supplementary Appendix 3 for details.

### Statistical analysis

Between-group differences in age, education, body mass index (BMI), total intracranial volume, HAMD score, and miR-146a-5p expression were assessed using a two-sample *t* test (or a Mann–Whitney *U* test for nonnormally distributed data). Between-group differences in sex and positive family history were evaluated using the χ^2^ test. Longitudinal changes in the HAMD scores of the MDD patients before and after treatment were assessed using the Wilcoxon signed-rank test. The threshold for statistical significance was set at *p* = 0.05.

Vertex-wise statistical analyses of cortical thickness were performed using the general linear model (GLM). The interaction effect of the factors ‘Depression’ (HC or diagnosed MDD patient) and ‘MiR-146a-5p’ (entered as a continuous variable) on cortical thickness was assessed using a ‘different-offset, different-slope’ (DODS) design. Here, the interaction effect of ‘Depression × MiR-146a-5p’ on cortical thickness is evaluated by testing the hypothesis that the correlation between cortical thickness and miR-146a-5p differs between MDD patients and HCs. A significant interaction effect indicates that the cortical thickness changes differently between MDD patients and HCs depending on the level of miR-146a-5p. A statistical significance threshold of *p* < 0.001 at vertex-level was used in combination with Monte Carlo null-*Z* simulation correction (10 000 iterations) for multiple vertex-level comparisons. Age, sex, education, and total intracranial volume were added as nuisance covariates to regress out their confounding effects. For each resulting cortical cluster with a significant interaction effect, the mean thickness was extracted and assessed for between-group differences to further determine whether the divergent tendencies of thickness change driven by miR-146a-5p could ultimately lead to significant thickness abnormalities in MDD patients compared with HCs. Moreover, the partial correlation between the mean thickness and HAMD score was performed for each cluster to further assess the behavioral relevance of observed cortical structural abnormalities (covariates: age, sex, education, total intracranial volume, and first-episode or recurrent depression; significance threshold: *p* < 0.05). For reference, the vertexwise difference in cortical thickness between the MDD and HC groups was also assessed using the GLM with a statistical significance threshold at vertex-level *p* < 0.001 combined with Monte Carlo null-*Z* simulation correction (10 000 iterations). To further assess the influences of the confounding factors on cortical thickness, linear regression was performed for each resulting region of interest (ROI) as a validation (See online Supplementary Appendix 4 for details).

To test whether the relationship between miR-146a-5p and brain structural abnormalities observed in MDD patients could be altered after antidepressant treatment, the correlation between baseline miR-146a-5p expression and cortical thickness of the significant cluster(s) after treatment was assessed., as was the correlation between baseline miR-146a-5p expression and the longitudinal thickness change of the cluster(s) before and after treatment. Partial correlation was used, controlling for age, sex, education, total intracranial volume, antidepressant dosage, and first-episode or recurrent depression (with *p* < 0.05 as the significance criterion). In addition, the significance of the longitudinal thickness change of the cluster(s) before and after treatment was assessed using the paired *t* test (with *p* < 0.05 as the significance criterion).

## Results

### Demographic & clinical characteristics

In total, 220 participants were enrolled in this study, comprising 113 MDD patients and 107 HCs (online Supplementary Fig. S1). There was no significant difference in age, sex, education, BMI, or total intracranial volume between the HC and MDD groups (all *p*s > 0.05, see Table 1). The HAMD score was significantly higher in MDD patients (*p* < 0.001). Among the MDD patients, the total course of depression prior to the study ranged from 0.5 to 276 months. Fifty-seven patients (50.4%) were drug-naïve MDD patients experiencing first-episode depression, and 56 MDD patients were experiencing a recurrent episode free of psychotropic medications (for a minimum of 3 months). Twenty-seven patients (23.0%) had a family history of psychiatric disorders.

**Table 1.** Demographic and clinical characteristics of patients with MDD and HCs

Characteristics	MDD (*n* = 113)	HCs (*n* = 107)	Statistics	*p* value
Age (years)	27 (18–64)	27 (18–64)	*Z* = −0.775	0.438
Sex (male/female)	35/78	32/75	χ^2^ = 0.030	0.864
Education (years)	14 (0–22)	13 (0–24)	*Z* = −0.216	0.829
Body mass index	20.55 (15.79–29.38)	21.48 (16.61–30.35)	*Z* = −1.842	0.066
Total intracranial volume ( × 10^6^ mm^3^)	1.42 ± 0.23	1.43 ± 0.21	*T* = −0.421	0.674
Total course of illness (months)	12 (0.5–276)	–	–	–
First-episode/recurrent depression	57/56	–	–	–
Family history of psychiatric disorder (percentage)	23.0%	1.9%	χ^2^ = 22.112	<0.001*
HAMD score	28 (17–52)	1 (0–11)	*Z* = −12.881	<0.001*

HAMD, Hamilton Rating Scale for Depression.

* indicates statistical significance; data are presented as the median (range) (or mean ± standard deviation when a parametric statistical test was used).

### Confirmation of circulating exosome isolation and expression of exosomal miR-146a-5p

**Fig. 1.**
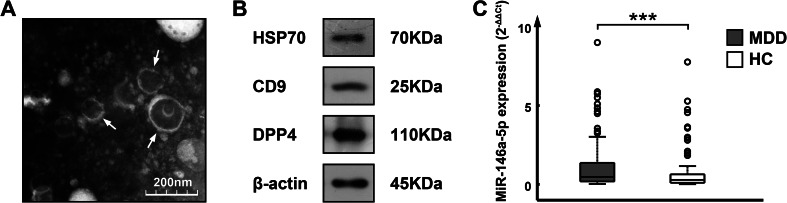
Expression of exosomal miR-146a-5p in serum samples from patients with MDD and HCs. (a) Serum-derived exosomes were visible on via transmission electron micrography as cup-shaped vesicles bound by bilayer membranes and measuring less than 200 nm in size (scale bar = 200 nm). (b) A western blot shows the positive exosomal markers HSP70, CD9, and DPP4 in exosome samples (*β*-actin served as a reference protein). (c) Exosomal miR-146a-5p expression as measured by qRT-PCR was significantly elevated in MDD patients compared with HCs (*p* = 0.001, Mann–Whitney *U* test).

Exosomes derived from the serum of MDD patients and HCs were visualized with transmission electron microscopy (Fig. 1a). Vesicles that morphologically resembled exosomes were identified as cup-shaped vesicles that had bilayer membranes and measured less than 200 nm in size. Western blot analysis confirmed positive expression of three exosomal markers, namely, HSP70, CD9, and DPP4 (Fig. 1b and online Supplementary Fig. S2).

The average Ct values from the quantitative reverse transcriptase polymerase chain reaction (qRT-PCR) analysis of miR-146a-5p, ranging from 24.06 to 30.05 for all the participants, indicated successful recovery of miR-146a-5p from the circulating exosomes. The 2^−ΔΔCt^ value was significantly increased in MDD patients (median, 0.47; range, 0.02–13.55) relative to HCs (median, 0.28; range, 0.01–7.76) (*Z* = −3.205, *p* = 0.001, Fig. 1c).

### Interaction effect of ‘Depression × MiR-146a-5p’ on cortical thickness

To determine whether the cortical thickness was altered depending on the expression level of miR-146a-5p, the interaction effect of ‘Depression × MiR-146a-5p’ was evaluated. It was noted that the raw miR-146a-5p data significantly followed a nonnormal distribution (*p* < 0.001 by Kolmogorov-Smirnov test). Therefore, to avoid bias caused by extreme values, a log transformation was performed before vertexwise GLM analysis. After log transformation, the miR-146a-5p data of both the MDD and HC groups were normally distributed.

**Fig. 2.**
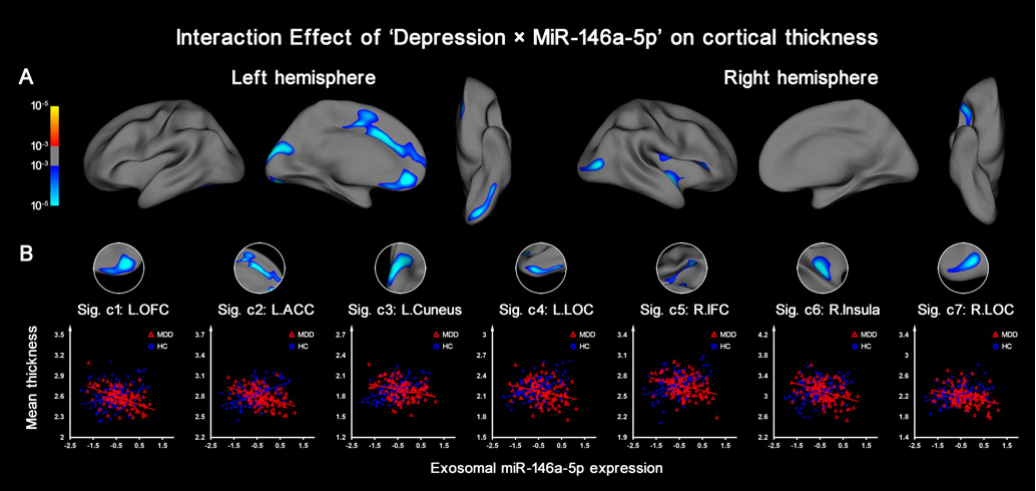
Vertex-wise statistical evaluation of the ‘Depression × MiR-146a-5p’ interaction effect on cortical thickness. (a) Cortical regions showing a significant ‘Depression × MiR-146a-5p’ interaction effect on cortical thickness are indicated (vertex-level *p* < 0.001 with Monte Carlo simulation correction). (b) For each cluster with a statistically significant interaction effect, the divergent correlation tendencies between cortical thickness and miR-146a-5p expression are shown separately for the two groups: HCs and patients with MDD.

Vertexwise GLM analysis revealed seven cortical regions for which the effect of depression on thickness was significantly dependent on the expression of miR-146a-5p (vertex-level *p* < 0.001 with Monte Carlo simulation correction): the left orbitofrontal cortex (OFC), the left anterior cingulate cortex (ACC), the left cuneus, the right inferior frontal cortex (IFC), the right insula, and the bilateral lateral occipital cortices (LOCs) (Table 2, Fig. 2a).

**Table 2.** Cortical areas showing a significant ‘Depression × MiR-146a-5p’ interaction effect on thickness

				Between-group difference		
Cortical areas	Maximum statistic	Cluster size (mm^2^)	Mean thickness in MDD	Mean thickness in HCs	*F* value	*p* value
L. OFC	6.875	769	2.58 ± 0.15	2.61 ± 0.18	4.745	0.030*
L. ACC	6.464	1776	2.80 ± 0.13	2.84 ± 0.15	10.101	0.002*
L. Cuneus	6.015	1146	1.89 ± 0.13	1.90 ± 0.13	0.596	0.441
L. LOC	5.316	1052	2.16 ± 0.16	2.22 ± 0.15	7.777	0.006*
R. IFC	4.876	681	2.65 ± 0.15	2.68 ± 0.16	3.493	0.063
R. Insula	5.918	490	3.09 ± 0.20	3.11 ± 0.21	3.221	0.074
R. LOC	5.244	561	2.19 ± 0.18	2.25 ± 0.17	5.379	0.021*

L., left; R., right; ACC, anterior cingulate cortex; OFC, orbitofrontal cortex; LOC, lateral occipital cortex; IFC, inferior frontal cortex.

* indicates statistical significance in between-group comparisons; thickness data are presented as the mean ± standard deviation for the MDD group and the HC (HC) group.

Then, the mean thickness values were further extracted from the above seven clusters with significant interaction effects (i.e. the ROIs). The interaction graph revealed divergent correlation tendencies between the two groups: higher miR-146a-5p expression was significantly related to thinner cortex in MDD patients but not in HCs (Fig. 2b). Moreover, among the seven clusters, the left ACC, OFC, and bilateral LOCs were found to have significant cortical thinning in the MDD group compared with the HC group (*p* < 0.05), while no significant between-group differences were found in the remaining three clusters (Table 2). Moreover, a vertexwise between-group comparison of cortical thickness is shown in online Supplementary Fig. S3 for a comprehensive reference. Plus, detailed results of the assessment of the confounding factors were summarized in online Supplementary Appendix 4 and Table S2.

### Correlations of cortical thickness with miR-146a-5p expression and HAMD

The mean thickness of the seven identified cortical clusters with significant ‘Depression × MiR-146a-5p’ interaction was further correlated with miR-146a-5p expression and HAMD score (for the MDD group only). Before correlation analyses, a log transformation was performed for the miR-146a-5p expression data to increase the normality of the data. All seven clusters were found to have significant negative correlations between thickness and miR-146a-5p expression (Table 3 and online Supplementary Fig. S4a). A significant negative correlation between HAMD scores and cortical thickness was found in the left cuneus (*r* = −0.209, *p* = 0.030). However, for the other six clusters, the correlations between cortical thickness and HAMD scores were not significant (online Supplementary Fig. S4b).

**Table 3.** Correlations of miR-146a-5p expression with cortical thickness and longitudinal thickness change before and after antidepressant treatment

	Baseline		Treated		Longitudinal change	
Cortical areas	*r*	*p* value	*r*	*p* value	*r*	*p* value
L. OFC	−0.494	<0.001*	−0.334	0.089	0.384	0.048*
L. ACC	−0.448	<0.001*	−0.323	0.100	0.059	0.768
L. Cuneus	−0.342	<0.001*	−0.215	0.283	0.303	0.125
L. LOC	−0.290	0.002*	−0.150	0.455	0.325	0.098
R. IFC	−0.327	0.001*	0.035	0.862	0.202	0.311
R. Insula	−0.381	<0.001*	−0.316	0.109	0.232	0.244
R. LOC	−0.337	<0.001*	−0.223	0.264	0.493	0.009*

L., left; R., right; ACC, anterior cingulate cortex; OFC, orbitofrontal cortex; LOC, lateral occipital cortex; IFC, inferior frontal cortex.

* indicates statistical significance. Note: Only patients with depression were included in the correlation analyses, and 33 patients who completed the follow-up were included in the correlation analysis after antidepressant treatment.

### Relationship between miR-146a-5p and cortical thickness after antidepressant treatment

To test whether the relationship between miR-146a-5p and brain structural variation in MDD patients could be altered after antidepressant treatment, we assessed the correlation between baseline miR-146a-5p expression and cortical thickness in the seven ROIs after treatment, as well as the correlation between baseline miR-146a-5p expression and longitudinal thickness change in the seven ROIs before and after treatment. Thirty-three MDD patients completed the follow-up and were included in the follow-up analysis (online Supplementary Fig. S1). A log transformation was performed for the data of miR-146a-5p expression to increase the normality of the data.

After treatment, the HAMD score of the MDD cohort dropped by 5 to 42 (*T* = 14.895, *p* < 0.001), indicating responsiveness to the treatment. Among the seven clusters with a significant ‘Depression × MiR-146a-5p’ interaction effect, a significant increase in cortical thickness after treatment was noted only in the right LOC (*T* = 2.886, *p* = 0.007) (online Supplementary Fig. S5a). The correlations between baseline miR-146a-5p expression and cortical thickness after treatment were found to be nonsignificant for all seven clusters. A significant positive correlation between baseline miR-146a-5p expression and longitudinal thickness change before and after treatment was found in the left OFC (*r* = 0.384, *p* = 0.048) and in the right LOC (*r* = 0.493, *p* = 0.009), indicating that higher miR-146a-5p expression was related to more remarkable cortical thickening after treatment (Table 3, online Supplementary Fig. S5b, and see the schematic plot in online Supplementary Video S1 for an illustration).

## Discussion

This study revealed a distinct association of exosomal miR-146a-5p with the cortical thickness pattern in patients with MDD compared with healthy individuals, i.e., overexpression of miR-146a-5p was associated with reduced cortical thickness in the left ACC, left OFC, bilateral LOC, and other regions in the depressed brain. Moreover, this relationship disappeared in the patients who received antidepressant treatment. Collectively, these results may indicate a process of miR-146a-5p-driven cortical atrophy in patients with MDD and the regions of the ACC, OFC, etc., may serve as important target cortical regions for the effect of miR-146a-5p.

First, the cortical distribution of the regions that showed a significant association with miR-146a-5p in this study was largely consistent with that of the regions identified in the literature as ‘hotspots’ of structural abnormalities in patients with MDD (Lee et al., 2021; Schmaal et al., 2017; Serra-Blasco et al., 2021). Specifically, in three recent meta-analysis studies (each with a sample size of over 1000 MDD patients), the left OFC (Schmaal et al., 2017; Serra-Blasco et al., 2021; Suh et al., 2019), left ACC (Schmaal et al., 2017; Serra-Blasco et al., 2021), right insula (Schmaal et al., 2017; Serra-Blasco et al., 2021), and right IFC (Serra-Blasco et al., 2021) were highlighted as convergent cortical regions showing GM reduction in patients with MDD in comparison with HCs. In particular, the OFC and ACC are intensely involved in emotion and social function regulation, and their abnormalities are considered to be related to an array of behavioral deficits in MDD (Peng et al., 2021; Zhao et al., 2017). Therefore, the cortices showing miR-146a-5p-dependent atrophy, as observed in this study, overlap with the classic cortices of abnormalities in MDD, further confirming that miR-146a-5p may play a role in mediating brain structural abnormalities in MDD. On the other hand, the occipital cortices (bilateral LOCs and cuneus cortex), which were also identified to have a significant relationship with miR-146a-5p in our results, are less frequently reported in the literature; this discrepancy could be important. Additionally, the correlation between HAMD scores and cortical thickness in the cuneus further supported the clinical relevance of occipital disruption in our results. Despite the discrepancy, it is worth noting that previous evidence also supported the involvement of the LOC in emotional information processing (Geerligs, Cam, & Henson, 2016), and occipital abnormalities could contribute to the emotional dysfunction of MDD (Chen et al., 2016, 2019), especially joint deficits of the LOC and OFC in fronto-occipital disconnection (Alders et al., 2019). Taken together, these findings support the roles of the ACC, OFC, occipital cortex, etc., as important cortical targets for the mediating effect of miR-146a-5p in MDD and may imply a complex mediation process.

The relationship between miR-146a-5p and changes in GM volume as observed in this study is supported by a series of cellular/molecular evidence. Previous studies have revealed that exosomal miR-146a-5p of microglial origin is exported to neurons (Fan et al., 2022; Prada et al., 2018), decreases the expression of KLF4 and Cdkl5, suppresses the formation of neurons and induces depression-like behaviors (Fan et al., 2022; Fuchs et al., 2014). Similarly, another study showed that miR-146a-5p could induce synaptic alterations, influencing dendritic spine formation and synaptic stability, via an inflammatory process mediated by presynaptic synaptotagmin1 (Syt1) and postsynaptic neuroligin1 (Nlg1) (Prada et al., 2018). In addition, morphological analysis provides direct evidence in that prolonged exposure to overexpressed miR-146a-5p leads to a significant decrease in dendritic spine density (Prada et al., 2018), which underlies volumetric loss of GM (Anderson, 2011). Taken together, all the evidence is in agreement with our findings and confirms the relationship between elevated miR-146a-5p and decreased GM volume in the depressed brain, contributes to the pathogenesis of depression. However, these speculations on the molecular/cellular means by which miR-146a-5p acts in the human brain are largely hypothetical. In particular, the described processes related to neurogenesis are largely based on the hippocampus in animal models. Although evidence has indicated that neural precursor cells could be isolated in human cortex, thus supporting the role of the cortex in neurogenesis (Arsenijevic et al., 2001), the relevant experiment was not replicated in this study. Therefore, the above speculations should be taken with caution.

The interplay between miR-146a-5p and antidepressant treatment has attracted a great deal of interest in recent years (Enatescu et al., 2016; Kim et al., 2019; Lopez et al., 2017a). In this study, repeated MRI was performed to establish whether the underlying pathological relationship between miR-146a-5p and cortical atrophy in MDD patients could be inhibited or reversed by antidepressants. Finally, the nonsignificant correlation between miR-146a-5p and cortical thickness after treatment, together with the significant relationship between miR-146a-5p overexpression and longitudinal cortical thickening after treatment in part of the cortices, might indicate that antidepressant treatment inhibits (or at least weakens) the effect of miR-146a-5p on cortical atrophy (see online Supplementary Video S1 for an illustration). Conversely, the notion that the treatment-related effect on cortical thickness is mediated by miR-146a-5p might also be supported. These longitudinal results provide evidence in addition to the cross-sectional results in this study, and both sets of findings serve as evidence that the statistical relationship between miR-146a-4p overexpression and cortical atrophy as currently observed is probably not accidental but pathologically relevant. However, it should be noted that the longitudinal change in cortical thickness before and after treatment was nonsignificant in six of the seven observed ROIs. This evidence may be consistent with a recent meta-analysis showing that previously published findings regarding antidepressant-related structural changes in GM have been heterogeneous, as only a minority of the studies reported a significant volume increase after treatment (Enneking, Leehr, Dannlowski, & Redlich, 2020). Specific to our study, first, the data might be limited by the comparatively small longitudinal sample size as well as the short follow-up time, which may have been insufficient to yield detectable macro-level structural changes. Second, it is possible that the expected thickness increase in MDD patients was disturbed by the heterogeneity in miR-146a-5p expression, since the ROIs in the observation were drawn based on the ‘Depression × MiR-146a-5p’ interaction effect. Therefore, more studies are still needed to further investigate the specific mechanisms that realize the interplay among antidepressant treatment, miR-146a-4p, and cortical morphology in MDD.

The strengths of this study include a comparatively large sample size of medication-free patients, isolation of blood-derived exosomes, and repeated MR scans in a cohort for validation. However, several limitations of this study should be noted. First, the comparatively small sample size for longitudinal data as well as the lack of a replication cohort might limit the generalizability of the results. Similarly, miRNA quantification from blood samples taken at a single time point may influence the stability of the results. Second, despite the follow-up evidence supporting its clinical relevance, the relationships among miR-146a-4p, depression, and cortical atrophy were mainly based on statistical dependency and should not be interpreted as cause-effect relationships. The specific mechanism of the underlying mediation process remains to be investigated. Thus, the current results should be interpreted with caution. Third, although a previous study confirmed in a model of depression that exosomal miR-146a-5p in serum is mainly of microglial origin in the peripheral blood, the lack of direct evidence to support the neural origin of exosomal miR-146a-5p in this study should be considered another limitation. Moreover, it should be noted that this study did not collect exhaustive records of lifestyle factors and medical history (e.g. scoring of head injury), which may be related to the physical and psychological health of the participants. Accordingly, the possible confounding effects cannot be completely excluded.

For future research, we encourage more mechanistic studies in order to better specify the cellular/molecular basis on which miR-146a-5p-driven brain atrophy is linked to the pathology of MDD. In particular, based on ultra-high-resolution imaging modalities, investigation of the hippocampus as a key region in extensive animal experiments will be extremely helpful to link *in vivo* imaging findings with laboratory findings. Moreover, the specific pattern of antidepressant involvement in the process of miR-146a-5p-driven cortical atrophy should be investigated to promote the development of novel therapeutic approaches targeting miR-146a-5p.

## Conclusion

In summary, this study reveals that overexpression of exosomal miR-146a-5p is associated with reduced cortical thickness in patients with MDD. The evidence confirms the relationship between miR-146a-5p dysregulation and cortical atrophy in the ACC, OFC, etc. Therefore, these findings may support the mediating effect of miR-146a-5p dysregulation on brain cortical structure in patients with MDD and help clarify the specifics of this *in vivo* effect by identifying the possible target cortices in the human brain.

## Supplementary material

For supplementary material accompanying this paper visit https://doi.org/10.1017/S0033291722003567. click here to view supplementary material
