# Supplementary material for: Reduced cerebral cortex thickness is related to overexpression of exosomal miR-146a-5p in medication-free patients with major depressive disorder
Source: Psychol Med. 2022 Nov 25;53(13):6253–60. doi: 10.1017/S0033291722003567 (PMC10520590; doi:10.1017/S0033291722003567)
Supplement: Supplementary file 1 [file S0033291722003567sup.zip › S0033291722003567sup001.doc]

**Reduced cerebral cortex thickness is related to overexpression of exosomal miR-146a-5p in medication-free patients with major depressive disorder**

Yanjia Deng^1, 2^ ^†^, Ping Gong^1, †^, Shuguang Han^2^, Jingyu Zhang^3^, Shuai Zhang^2^, Bin Zhang^4^, Yong Lin^5, 6^, Kai Xu^2^, Ge Wen^3, *^, Kai Liu^1, 2, *^

**Supplementary Appendix**

**1. Isolation and verification of circulating exosomes**

To exclude the influence of medication and ensure consistency with MR data, venous blood samples were collected from each participant within 24 hours before or after the MR examination. Banking and handling of the blood samples followed the BRISQ (Biospecimen Reporting for Improved Study Quality) guidelines (see Table S1 for Tier 1 characteristics). Collected blood samples were kept at room temperature for half an hour, then centrifuged at 3,000 × g for 15 min. Separated aliquots of serum were used for exosomal miR-146a-5p isolation. First, 1 mL of serum was centrifuged at 2,000 × g for 20 min to remove cellular organelles and other cell debris. Second, the serum was mixed with Ribo^TM^ exosome isolation reagent (for plasma or serum; RIBOBIO, China), incubated at 4°C for 30 min, and then centrifuged at 15,000 × g for 2 min. The final supernatant containing the exosomes was transferred to a new tube for microRNA extraction.

The extracted exosome samples were authenticated by transmission electron microscope examination and Western blotting. Briefly, 10 μL of the exosome suspension was placed on 300-mesh carbon-coated copper grids for 10 min. The excess suspension was blotted away with filter paper. The exosomes were then negatively stained by placing the grids onto 10 μL of 2% uranyl acetate solution for 5 min and air dried. Finally, the samples were examined with a transmission electron microscope (HITACHI H-7650, Tokyo, Japan).

The exosome samples from 32 randomly selected participants were subjected to Western blots using primary antibodies (Abcam ab5181606 1:500, Abcam ab92726 1:500, and Abcam ab129060 1:500) against the exosomal surface markers HSP70, CD9, and DPP4, then washed with PBST. β-Actin served as a reference protein. Then, the membranes were incubated with secondary antibodies at a 1:500 dilution and washed again with PBST. The blots were developed with chemiluminescent reagents from Pierce (Thermo Fisher Scientific, USA).

**2. RNA extraction and quantification**

Total RNA was extracted from exosome preparations using TRIzol Reagent (Invitrogen, CA, USA) according to the manufacturer’s instructions. The quality of the RNAs was verified with a microspectrophotometer (KAIAO K2800, Beijing, China) by ensuring that the OD260/OD280 ratio exceeded 1.8, a standard criterion. Reverse transcription was performed using Superscript III RT (Invitrogen, USA) and random primers. The reverse transcription reaction product was immediately stored at -20°C for further analysis. Quantitative reverse transcriptase polymerase chain reaction (qRT‒PCR) was conducted with a set of primers to amplify miR-146a-5p (5′-CGCGTGAGAACTGAATTCCA-3′; 5′-AGTGCAGGGTCCGAGGTATT-3′) and the endogenous reference RNU6B (U6 , 5′-CTCGCTTCGGCAGCACA-3′; 5′-AACGCTTCACGAATTTGCGT-3′), which were designed online using Primer 5. The reaction mixture consisted of 10 μL of SYBR® *Premix Ex Taq*™ (Tli RNase H Plus, Japan) (2×), 5 μL of cDNA, 0.5 μL of reverse primer, 0.5 μL of forward primer, and 4 μL of RNase-free water. The reaction mixtures were incubated at 95°C for 5 min, followed by 39 cycles at 95°C for 10 s and 60°C for 34 s. The melting curve was determined at the end of the PCR cycles over a range of 60-95°C. All reactions were run in triplicate for each participant. The relative expression of miR-146a-5p was calculated by measuring the value of the cycle threshold change (ΔCt), i.e., the 2^-ΔΔCt^ method, with U6 as the endogenous control .

**3. MR image preprocessing**

The preprocessing of the 3D-T1W imaging data followed the standard *recon-all* stream of FreeSurfer (version 6.0.0, surfer.nmr.mgh.harvard.edu). The major steps included intensity inhomogeneity correction, automated Talairach transformation, skull stripping, brain tissue segmentation , tessellation of the gray matter (GM)–white matter (WM) boundary, automated topology correction, and surface deformation following intensity gradients to optimally place the GM–WM and GM–cerebrospinal fluid borders at the locations with the greatest intensity shifts . These steps generated the WM and pial surfaces for each individual brain, and the cortical thickness at each vertex on the pial surface was calculated as the average distance between the GM–WM boundary and the pial surface. For the surface-based vertexwise statistical analysis, inter-subject surface registration was performed by registering the cortical surface with the depths of gyri and sulci normalized to an averaged spherical surface , which was followed by spatial smoothing with a 15 mm full width at half maximum (FWHM) Gaussian kernel.

To ensure quality, the processed data of all the participants were manually inspected for any inaccuracies in Talairach transformation, skull stripping, or segmentation. GM–WM and pial boundaries were visually checked slice by slice, and if needed, manual delineation (e.g., the addition of control points) was performed for correction. Herein, this manual editing step was performed by personnel who were blinded to the grouping information of the participants. For most subjects, only minor edits were required after automatic preprocessing (one subject with widespread segmentation inaccuracies caused by excessive head motion during the MR scan was excluded; see Fig. S1).

**4. Regression analysis for confounding factors**

To further assess the influences of the confounding factors on cortical thickness, linear regression was performed for each resulting region of interest (ROI), using both main factors and confounding factors (i.e., age, sex, education, total intracranial volume, BMI, family history of psychiatric disorder, marital status, smoking, and occupation) as independent variables and cortical thickness in the ROI as a dependent variable. Stepwise linear regression was performed, with the significance levels for entry and removal being 0.05 and 0.10, respectively. The contributions of these confounding factors to the cortical thickness in the established models were assessed.

Among the confounding factors, age, sex, total intracranial volume, and marital status were found to have a significant influence on cortical thickness in at least one ROI after stepwise regression (Table S2). Then, to exclude the possible influence of the factor of marital status, which was not added as a covariate in previous steps of analysis, we additionally evaluated the ‘Depression × MiR-146a-5p’ interaction effect on cortical thickness in the bilateral LOC by adding marital status as a covariate. The results showed that the ‘Depression × MiR-146a-5p’ interaction effect on cortical thickness was still significant in both the left (*F* = 10.784, *P* = 0.001) and right (*F* = 12.072, *P* = 0.001) LOC. Therefore, these data supported that thickness variation driven by the ‘Depression × MiR-146a-5p’ interaction effect is independent of marital status.

**References:**

D'Haene, B., Mestdagh, P., Hellemans, J., & Vandesompele, J. (2012). miRNA expression profiling: from reference genes to global mean normalization. *Methods in Molecular Biology*, *822*, 261-272. doi: 10.1007/978-1-61779-427-8_18

Fischl, B., Salat, D. H., Busa, E., Albert, M., Dieterich, M., Haselgrove, C., . . . Dale, A. M. (2002). Whole brain segmentation: automated labeling of neuroanatomical structures in the human brain. *Neuron*, *33*(3), 341-355. doi: 10.1016/s0896-6273(02)00569-x

Fischl, B., Sereno, M. I., & Dale, A. M. (1999). Cortical surface-based analysis. II: Inflation, flattening, and a surface-based coordinate system. *Neuroimage*, *9*(2), 195-207. doi: 10.1006/nimg.1998.0396

Livak, K. J., & Schmittgen, T. D. (2001). Analysis of relative gene expression data using real-time quantitative PCR and the 2(-Delta Delta C(T)) *Method. Methods*, *25*(4), 402-408. doi: 10.1006/meth.2001.1262

Moore, H. M., Kelly, A. B., Jewell, S. D., McShane, L. M., Clark, D. P., Greenspan, R., . . . Vaught, J. (2011). Biospecimen reporting for improved study quality (BRISQ). *Cancer Cytopathology*, *119*(2), 92-101. doi: 10.1002/cncy.20147
