# Supplementary material for: Reduced cerebral cortex thickness is related to overexpression of exosomal miR-146a-5p in medication-free patients with major depressive disorder
Source: Psychol Med. 2022 Nov 25;53(13):6253–60. doi: 10.1017/S0033291722003567 (PMC10520590; doi:10.1017/S0033291722003567)
Supplement: Supplementary file 1 [file S0033291722003567sup.zip › S0033291722003567sup002.doc]

**Supplementary Tables**

**Table S1. BRISQ checklist for blood sample handling and banking: Tier 1 items**

|  | Data Elements |  |
| --- | --- | --- |
| ❒ | **Biospecimen type** | Serum |
|  | *Solid tissue, whole blood, or another produce derived from human being* | |
| ❒ | **Anatomical site** | Antecubital area of the arm |
|  | *Organ of origin or site of blood draw* | |
| ❒ | **Disease status of patients** | Major depressive disorder, Healthy control |
|  | *Controls or individuals with the disease of interest* | |
| ❒ | **Clinical characteristics of patients** |  |
|  | *Available medical information known or believed to be pertinent to the condition of the biospecimens* | |
| ❒ | **Vital State of patients** | Alive |
|  | *Alive or deceased patient when biospecimens were obtained* | |
| ❒ | **Clinical diagnosis of patients** | Major depressive disorder |
|  | *Patient clinical diagnoses (determined by medical history, physical examination, and analyses of the biospecimen) pertinent to the study* | |
| ❒ | **Pathology diagnosis** | NA |
|  | *Patient pathology diagnoses (determined by macro and/or microscopic evaluation of the biospecimen at the time of diagnosis and/or prior to research use) pertinent to the study* | |
| ❒ | **Collection mechanism** | Peripheral blood draw |
|  | *How the biospecimens were obtained* | |
| ❒ | **Type of stabilization** | kept at room temperature for half an hour, followed by centrifugation at 3,000 × g for 15 min |
|  | *The initial process by which biospecimens were stabilized during collection* | |
| ❒ | **Type of long-term preservation** | Freezing |
|  | *The process by which the biospecimens were sustained after collection* | |
| ❒ | **Constitution of preservative** | NA |
|  | *The make-up of any formulation used to maintain the biospecimens in a non-reactive state* | |
| ❒ | **Storage temperature** | −80 °C |
|  | *The temperature or range thereof at which the biospecimens were kept until distribution/analysis* | |
| ❒ | **Storage duration** | 1 month to 1.5 year |
|  | *The time or range thereof between biospecimen acquisition and distribution or analysis.* | |
| ❒ | **Shipping temperature** | NA |
|  | *The temperature or range thereof at which biospecimens were kept during shipment or relocation.* | |
| ❒ | **Composition assessment & selection** | OD260/OD280 ratio > 1.8 for extracted RNA sample; Original Ct values between 26 to 35 in all of the three qRT-PCR*s* |
|  | *Parameters used to choose biospecimens for the study* | |

BRISQ = Biospecimen Reporting for Improved Study Quality

**Table S2. Influences of the confounding factors on cortical thickness of the seven regions with significant interaction effect (‘Depression × MiR-146a-5p’)**

| Dependent  Independent | L. OFC | | L. ACC | | L. Cuneus | | L. LOC | | R. IFC | | R. Insula | | R. LOC | |
| --- | --- | --- | --- | --- | --- | --- | --- | --- | --- | --- | --- | --- | --- | --- |
|  | *β* | *P* | *β* | *P* | *β* | *P* | *β* | *P* | *β* | *P* | *β* | *P* | *β* | *P* |
| Age | -0.205 | 0.006* | -0.429 | <0.001* | -0.139 | 0.063 | - | 0.402 | -0.397 | <0.001* | -0.448 | <0.001* | - | 0.632 |
| Sex | - | 0.464 | 0.210 | 0.002* | - | 0.998 | - | 0.998 | - | 0.861 | - | 0.676 | 0.166 | 0.034* |
| Education | - | 0.731 | - | 0.861 | 0.106 | 0.142 | - | 0.452 | - | 0.481 | - | 0.404 | - | 0.436 |
| Body mass index | -0.096 | 0.121 | - | 0.587 | - | 0.822 | - | 0.421 | - | 0.478 | 0.070 | 0.239 | - | 0.830 |
| Total intracranial volume | - | 0.938 | 0.073 | 0.283 | -0.207 | 0.002* | - | 0.530 | - | 0.507 | - | 0.368 | 0.112 | 0.154 |
| Family history of psychiatric disorder | - | 0.530 | -0.088 | 0.115 | -0.083 | 0.225 | - | 0.446 | - | 0.370 | - | 0.713 | -0.103 | 0.127 |
| Marriage | - | 0.423 | - | 0.908 | - | 0.559 | -0.178 | 0.007* | - | 0.729 | 0.084 | 0.159 | -0.172 | 0.012* |
| Cigarette use | - | 0.873 | 0.064 | 0.246 | - | 0.502 | 0.065 | 0.305 | 0.073 | 0.215 | - | 0.847 | - | 0.877 |
| Occupation | 0.098 | 0.172 | - | 0.963 | - | 0.975 | 0.105 | 0.097 | - | 0.844 | 0.122 | 0.076 | - | 0.862 |

L. = left, R. = right; ACC = anterior cingulate cortex, OFC = orbital frontal cortex, LOC = lateral occipital cortex, IFC = inferior

frontal cortex. Note: Stepwise regression analysis is performed, and the statistics of the model with largest adjusted *R*^2^ are listed; Standardized *β* is used; * indicates the independent variable with statistical significance (*P* <0.05); Cigarette use refers to the daily consumption of cigarettes (rating from 0-4, respectively corresponding to ≤10, 11-20, 21-30, and ≥31); Occupation is classified according to manual and mental labour.

**Supplementary Figures**

**Figure S1**. Flow diagram for participants exclusion of patients with major depressive disorder (MDDs) and healthy controls (HCs).

**Figure S2.** Western blot analysis shows the expression of positive exosomal markers HSP70, CD9, and DPP4 for all thirty-two randomly selected participants.

**Figure S3**. Vertexwise comparison of cortical thickness between patients with major depressive disorder (MDDs) and healthy controls (HCs) estimated by FreeSurfer. The statistical map is thresholded with a vertex-level *P* < 0.001. Compared with HC group, MDD group showed significantly lower cortical thickness in the left superior frontal gyrus, left anterior cingulate cortex, left orbital frontal cortex, left fusiform gyrus, left lateral occipital cortex, left middle/inferior temporal cortices, right middle/inferior cortices, right fusiform gyrus, right lateral occipital cortex, and right superior/middle temporal cortices.

**Figure S4**. Correlations of the thickness (extracted from the seven significant clusters identified as having a significant interaction effect of ‘Depression × MiR-146a-5p’) to miR-146a-5p expression and to Hamilton Rating Scale for Depression (HAMD) score. Partial correlation is used, controlling for age, sex, education, total intracranial volume, and first-episode or not (* indicates *P* < 0.05). Note: Only patients with depression are included for the correlation analyses.

**Figure S5.** Comparisons of cortical thickness in clusters with a significant ‘Depression × MiR-146a-5p’ interaction effect before and after antidepressant treatment (A) and correlations between the longitudinal thickness change and baseline miR-146a-5p expression (B).
